# Who could be One Health Activist at the community level?: A case for India

**DOI:** 10.1186/s12960-021-00558-3

**Published:** 2021-01-22

**Authors:** Sandul Yasobant, Walter Bruchhausen, Deepak Saxena, Farjana Zakir Memon, Timo Falkenberg

**Affiliations:** 1 Center for Development Research (ZEF), University of Bonn, Genscherallee 3, 53113 Bonn, Germany; 2 Global Health, Institute for Hygiene and Public Health (IHPH), University Hospital Bonn, 53127 Bonn, Germany; 3 Indian Institute of Public Health Gandhinagar (IIPHG), Gandhinagar, 382042 India; 4 Jawaharlal Nehru Medical College, Datta Meghe Institute of Medical Sciences (DMIMS), Wardha, 442004 India; 5 GeoHealth Centre, Institute for Hygiene and Public Health (IHPH), University Hospital Bonn, 53127 Bonn, Germany

**Keywords:** CHW, Motivation, ASHA, OHA, One Health, India

## Abstract

**Background:**

Community health workers (CHWs) are the mainstay of the public health system, serving for decades in low-resource countries. Their multi-dimensional work in various health care services, including the prevention of communicable diseases and health promotion of non-communicable diseases, makes CHWs, the frontline workers in their respective communities in India. As India is heading towards the development of One Health (OH), this study attempted to provide an insight into potential OH activists (OHA) at the community level. Thus, this case study in one of India’s western cities, Ahmedabad, targeted identifying OHA by exploring the feasibility and the motivation of CHWs in a local setting.

**Methods:**

This case study explores two major CHWs, i.e., female (Accredited Social Health Activists/ASHA) health workers (FHWs) and male (multipurpose) health workers (MHWs), on their experience and motivation for becoming an OHA. The data were collected between September 2018 and August 2019 through a mixed design, i.e., quantitative data (cross-sectional structured questionnaire) followed by qualitative data (focus group discussion with a semi-structured interview guide).

**Results:**

The motivation of the CHWs for liaisoning as OHA was found to be low; however, the FHWs have a higher mean motivation score [40 (36–43)] as compared to MHWs [37 (35–40)] out of a maximum score of 92. Although most CHWs have received zoonoses training or contributed to zoonoses prevention campaigns, their awareness level was found to be different among male and female health workers. Comparing the female and male health workers to act as OHA, higher motivational score, multidisciplinary collaborative work experience, and way for incentive generation documented among the female health workers.

**Conclusion:**

ASHAs were willing to accept the additional new liaison role of OHAs if measures like financial incentives and improved recognition are provided. Although this study documented various systemic factors at the individual, community, and health system level, which might, directly and indirectly, impact the acceptance level to act as OHA, they need to be accounted for in the policy regime.

## Background

The health workforce’s skill and motivation directly influence the health system functionality across the globe [1, 2]. Evidence from low- and middle-income countries (LMICs) denoted a shortage of the health workforce, leading to gaps in service coverage and undermining the achievement of the health-related Sustainable Development Goals [3, 4]. According to the World Health Organization, 18 million additional health workers are needed to achieve universal health coverage by 2030 in LMICs [4]. One among other strategies is to address this shortfall through “task-shifting”, i.e., allocation of tasks to actors at the lowest level who can perform them successfully [5, 6]. In this context, the concept of using community health workers (CHWs) has gained acceptance again [7].

The umbrella term “community health worker” includes frontline functionaries to deliver patient-centric, comprehensive primary health care, address social determinants of health, and respond to various health challenges and outcomes at the community level [8]. With an intimate understanding of the respective communities, CHWs are frontline health workers who serve their community as liaisons between health/social services and the community [7, 9]. In promoting universal health coverage, these CHWs play a major role and have been deployed globally as a local, low-cost health resource in communities [10]. According to various country reports, the role of CHWs generally includes health promotion, disease prevention, treatment of basic medical conditions, and collection of health data [7, 11, 12]. In addition, CHWs also have been considered a valuable asset during outbreaks for social mobilization and the distribution of health information, thus improving health security and community-level resilience[13].

In LMICs, CHWs are fronting challenges in effective healthcare delivery not only for achieving universal health coverage [14], but also due to the several large outbreaks and (re) emerging diseases with an increased burden of zoonotic diseases [15]. While the One Health (OH) approach is emerging on the global agenda to tackle zoonotic (re-)emerging diseases, it also emphasizes the importance of a skilled workforce and intersectoral collaboration among human, animal, and environmental health sectors for its operationalization [16, 17]. So far, different countries have attempted to reinforce their health workforce to act collaboratively [18, 19], and few piloted combined human and animal health services [20]. Unpacking qualitative and quantitative problems in the skilled workforce becomes further challenging when intersectoral collaboration is promoted across the OH domains [21]. This is one reason why the current research was focused on investigating who could be a potential activist, liaised either for risk identification or disease control (or both) at the interface of the human–animal–environment in communities of India. This idea has primarily emerged for two reasons: first, the risk is not being identified in an intersectoral exchange or manner, which leads us to face uncontrolled epidemic or pandemic situations, and second, the disease control strategies in the human and animal sector are not being implemented uniformly. For example, India’s human health sector’s surveillance collects symptom-based information [22], whereas the animal health sector collects the only laboratory-confirmed diagnosed cases [23]. Therefore, there is an urgent need to identify the risks from the human–animal–environment interface at the community level in a comparable manner through health activists, who could be framed as “One Health Activist” (OHA). Due to its interdisciplinarity nature, these actors at the community level were considered activists rather than workers (supposed to be a formal health workforce). The assumption is that without this label, the OHAs should not automatically be assumed to be progressive [24]. Thus, authors assumed this workforce as activists rather than workers, although these activists could be promoted later as workers. Among these three domains (human, animal, and environmental health) in India, the health workforce has the maximum reach at the community level through the CHWs, including Accredited Social Health Activists (ASHAs), Multipurpose health workers (MPHW), and Anganwadi Workers (AWWs) [25]. In one of our previous studies under the RICOHA (Research to explore intersectoral collaboration for One Health approach) project [26], we have documented the absence of governmental community actors from the animal health system in urban India [27]. Other evidence also indicates an acute shortage of an animal health workforce both at the clinical and the community level over decades [28], therefore identifying actors from the health workforce, who already have a good reach at the community level, might provide an opportunity for smooth operationalization of OH activities at the grass-root level. Recognizing that CHWs already play a role in pandemic preparedness and represent a trusted voice in the community, these CHWs were examined for their potential role as OHA in one of the western cities of Gujarat state, Ahmedabad. According to the literature, there are diverse motivational factors that influence the work performance of these CHWs [10, 29], this particular case study attempted to explore the motivational factors to become an OHA at the community level.

### Objective(s)

1. To assess the awareness level of selected zoonotic diseases among the CHWs.
2. To document the multi-dimensional work pattern and performance of the CHWs.
3. To understand the level of motivation of the CHWs for becoming an OHA.

## Methods

### Study type

This case study used a mixed-method design. Quantitative data collection (cross-sectional survey) was followed by qualitative data collection (focused group discussion) from September 2018 to August 2019.

### Study setting

The study was conducted in one of the most populous cities of the western state of Gujarat, Ahmedabad. It is the seventh most populous city in India and is the largest city of the western state Gujarat [30]. The city is further divided into zones and wards for administrative purposes. About 1500 CHWs are working across the six zones and 64 wards of the city and serving to a population of 7,650,000 [31]. Each CHW (predominantly female) ideally caters to the average population of 1000–2500 in India’s urban setting [32]. This particular case study was carried out in two administrative zones of the city, i.e., East and South zones, with their 23 wards. The reason for the purposive selection of these two zones was the high population density, higher quantity of community healthcare workers, and higher risk of disease outbreaks.

### Study sample

CHWs are working as an interface between the community and the public health system. These CHWs have been engaged in disease awareness, promote good health practices, and help the community in accessing health services for decades. In this study, two types of CHWs were targeted, i.e., Accredited Social Health Activists (ASHAs) as a female health workers (FHWs) and malaria/multipurpose male health workers as a male health workers (MHWs) based on gender. Initially, ASHAs were devoted to reproductive health services and family planning [33], but with the recent assignment for non-communicable diseases, their roles were expanded to other public health domains [34]. MHWs were involved in controlling communicable diseases, including malaria, TB, leprosy, water- and vector-borne diseases, environmental sanitation, detection of disease outbreaks, and their control [35]. In Ahmedabad, especially in the study area, the ratio of FHWs to MHWs per ward was 23:3. For the quantitative survey, all FHWs (~ 500) and MHWs (~ 70) were approached to participate in the study, and those who provided consent for the study were included in the final sample. The response rate was 58% in the case of FHWs and 87% in the case of MHWs. Therefore, the final sample for the study was 349 CHWs (288 FHWs and 61 MHWs). For the qualitative study, to participate in the focus group discussion (FGD), participants were contacted during the quantitative survey. About one-third of the participants provided their consent and availability were invited to participate in the FGD. Upon deciding a date and place, about 2–4 FHWs per each ward accepted final invitation, among which one per each ward was selected randomly and 5–6 FHWs were grouped for each FGD, based on the geographic convenience. Thus, four FGDs were conducted among the FHWs in two zones of the city. For the FGDs among the MHWs, similar rules were applied to randomly recruit MHWs from each ward. As the number of MHWs was less, one FGD was conducted per zone, thus two FGDs were conducted for the MHWs.

### Study data collection

For the quantitative component, a structured, pilot-tested questionnaire in the vernacular language was used to collect information on the socio-professional details, training in zoonoses, knowledge, and practices about the selected zoonotic diseases, details on the previous collaborative work, perception about the required factors for becoming an OHA and the motivation level. The zoonotic diseases rabies, brucellosis, swine flu, and bird flu had been selected during the previously conducted prioritization workshop for Ahmedabad [36]. A standardized tool, validated in the Indian setting by Tripathy et al*.* [37] and originally developed by Bennet et al*.* [38], was used to measure the motivation. The motivation tool of Tripathy et al*. *[37] consists of 23 items with eight primary constructs, i.e., general motivation, burnout, job satisfaction, conscientiousness, timeliness, and personal issues. The responses were captured through an agreement scale of 1 to 4, i.e., strong disagreement (1) to a strong agreement (4). For negative questions, reverse coding was implemented before analysis. A trained research assistant administered the tool, which required 20–30 min of time from each participant.

FGDs were conducted for the qualitative component. The FGDs were conducted face-to-face at a time and place (mostly at the health centers) convenient to the participants, using an interview guide in the vernacular language. The empty hall of the health centers was utilized to conduct these FGDs, there was no other healthcare staff allowed to be present during the discussion, and the disclaimer was made to ensure the information confidentiality, which resulted in the improved degree of comfort and participation in the discussion. The interview guide focused on their current job tasks and the motivation for becoming an OH activist. Each FGD was conducted in the presence of the researcher and participants only and lasted over 1 h. At the end of each FGD, the major points of discussion were summarized for the participants based on the field notes. All FGDs were recorded with due consent from the participants.

### Study data analysis

For the quantitative component, data were entered in Epi-Info (7.2.3.1) and exported to EpiData Analysis (version 2.2.2.183) for analysis [39]. The descriptive statistics were segregated between the groups of FHWs and MHWs. Categorical variables were expressed as frequencies or percentages, whereas the continuous variable was expressed as means with standard deviation. The total motivation score for each respondent was computed by adding the individual agreement score of all 23 items. The minimum and maximum possible score of the tool was 23 and 92, respectively. The motivation score was expressed in the form of a mean score. To assess differences between these groups, Chi-square tests were used for categorical variables, and t-tests were applied for continuous variables.

For the qualitative component, the recordings and field notes were uploaded to Atlas.Ti (version 7.5.18) [40]. The recordings were transcribed for the development of the final transcripts. The transcripts were analyzed based on the previously decided themes, i.e., the current level of motivation and challenges at the individual, community, health system level, and the motivations for becoming an OH activist. The quantitative motivational score was compared and discussed with the qualitative findings.

## Results

### Quantitative findings

Out of the 349 CHWs sampled from two zones of the Ahmedabad city, 288 were FHWs, and 61 were MHWs, with a mean age of 40.38 ± 7.65, 36.25 ± 6.48, respectively. Although secondary education is the minimum qualification for the FHWs, we have documented that one-third of the sampled FHWs only completed primary education. Most of the MHWs were found to have at least a bachelor's degree. The professional experience in both categories was found to be similar: 8.29 ± 4.56, 8.40 ± 3.78 years, respectively. The mean catering population per FHWs was 3,183 ± 2,108, compared to the 47,718 ± 66,966 for the MHWs. The mean working hours per day were 4.49 ± 0.73 and 8.13 ± 0.81 for FHWs and MHWs, respectively. As the FHWs are on incentive-based working models, their mean income in INR was 4098 ± 1190, whereas MHWs are on salary-based models with mean incomes of 28,662 ± 6914. The detailed differences are presented in Table 1.

**Table 1:** Socio-professional details of the community health workers in Ahmedabad, India, during 2018–19

Profile	FHWs *n* = 288(%)	MHWs *n* = 61(%)
Age (in years)	40.38 ± 7.65	36.25 ± 6.48
Education		
Up to secondary	88 (30.6)	0
Secondary/higher	132 (45.8)	13 (21.3)
Graduate/above	68 (23.6)	48 (78.7)
Professional experience (in years)	8.29 ± 4.56	8.40 ± 3.78
Catering population	3183 ± 2108	47,718 ± 66,966
Monthly income (INR)	4098 ± 1190	28,662 ± 6914
Working hours per day	4.49 ± 0.73	8.13 ± 0 .81

Continuous variables are expressed as mean ± SD

*FHWs* Female Health Workers, *MHWs* Male Health workers, *INR* Indian rupee

#### Training and knowledge on zoonoses

Table 2 represents the awareness of the selected zoonotic diseases, which was higher overall among the FHWs as compared to the MHWs, except for the national program on rabies and brucellosis symptoms. Most of the FHWs were aware of the anti-rabies vaccination, which was reflected in their practice, such as the higher proportion of ARV counseling or ARC referral. Similarly, the higher awareness about the flu symptoms was reflected in the practices of FHWs, such as either providing basic medications or UHC referral in case of flu-like cases. Overall, a higher proportion of FHWs than MHWs was aware of at least one symptom of human rabies, influenza.

**Table 2:** Awareness and practices on selected zoonotic diseases among the community health workers in Ahmedabad, India, during 2018–19

Factors	FHWs *n* = 288 (%)	MHWs *n* = 61 (%)	*p*-value
Awareness			
Awareness about National Rabies control Programme	99 (34.4)	45 (73.8)	0.000*
Awareness about the influenza vaccine?	246 (85.4)	54 (88.5)	0.526
Awareness about anti-rabies vaccines	287 (99.7)	59 (96.7)	0.024*
Aware about at least one symptom of rabies	226 (86.6)	41 (67.2)	0.060
Aware about at least one symptom of brucellosis	3 (1.1)	3 (4.9)	0.034*
Aware about at least one symptom of flu	285 (98.9)	60 (98.3)	0.690
Practices			
Ever received zoonosis training	229 (79.5)	16 (26.2)	0.000*
Ever participated zoonosis campaigning	194 (67.6)	49 (80.3)	0.049*
What you do when you come across a case of a dog bite?			
Counsel for ARV	43 (14.9)	6 (9.8)	0.298
Refer to UHC/ARC	203 (70.5)	29 (47.5)	0.001*
Inform to FHS/MO	15 (5.2)	0	0.068
What you do when you come across a case of flu-like symptoms?			
Give basic medicines	117 (40.6)	1 (1.6)	0.000*
Refer to UHC	256 (88.9)	47 (77)	0.013*
Inform to FHS/MO	9 (3.1)	3 (4.9)	0.485

*FHWs* Female Health Workers, *MHWs* Male Health workers, *ARV* anti-rabies vaccine, *UHC* Urban Health center, *ARC* anti-rabies clinic, *FHS* Female Health Supervisor, *MO* Medical Officer

*p < 0.05 is considered as significant, derived from the Chi-squared test for the female and male health worker

#### Multi-dimensional work of CHWs and prerequisites for OHAs

Upon enquiring about their previous work experience with other sectors, it was found that about 55% of FHWs have worked with the Women and Child Department (especially with Anganwadi Workers) in their normal daily routine. About 10% have worked with the higher administrative authorities from various departments during health emergencies; whereas, only 6% of MHWs mentioned previous collaborative work with any other sectors. Most health workers (97%) said their involvement in outbreak management in the past, and 80% agreed to additional engagements other than their primary task. Table 3 presents the summarized required factors that are strongly agreed by both groups to become an OHA. An essential element needed was institutional support (indicating the top-down directives) from all respective sectors, followed by structured guidelines with specific roles and responsibilities. Further, both groups mentioned adequate training on zoonoses, and leadership skills will be required, followed by social skills. Health workers also mentioned that there should be specific objectives and they need to be trained on coordinated roles with a focus on building trust with other actors.

**Table 3:** Required factors for becoming an OH activist as expressed with ‘Strongly agree’ by the sampled health workers of Ahmedabad, India, during 2018–19

Factors	FHWs *n* = 288 (%)	MHWs *n* = 61 (%)	*p*-value
Training on coordinating roles	208 (72.5)	39 (63.9)	0.182
Relation between staff members	87 (30.4)	22 (36.1)	0.388
Knowledge and skills training	235 (81.9)	56 (91.8)	0.057
Individuals’ social skills	239 (83.6)	53 (86.9)	0.519
Trust with other actors/departments	216 (75.3)	54 (88.5)	0.024*
Specific objective	234 (81.5)	52 (85.2)	0.491
Conflict resolution authority	141 (49.3)	20 (32.8)	0.019*
Institutional support	279 (97.2)	59 (96.7)	0.835
Leadership skills	252 (87.8)	58 (95.1)	0.098
Structured guidelines	268 (93.4)	60 (98.4)	0.129

*FHWs* Female Health Workers, *MHWs* Male Health Workers

*p < 0.05 is considered as significant, derived from the Chi-squared test for the female and male health worker

#### Level of the motivation of the CHWs

Overall, the motivation score of FHWs was 40 (36–43), higher as compared to the MHWs 37 (35–40) out of a maximum score of 92. The mean motivational score was significantly different among these groups. The overall mean motivation score was 1.8 ± 0.2 higher among FHWs, compared to 1.6 ± 0.2 among MHWs. Both these groups were found to have low motivation and they sensed burnout in their daily routine work, as shown in Table 4. The general motivation was found to be low overall, with a slightly higher score of 2.1 ± 0.6 among the FHWs compared to a score of 1.9 ± 0.5 of the MHWs. The mean constructs of motivation scores such as job satisfaction, organization commitment, conscientiousness, timeliness, personal issues were found to be low (with a mean score of less than 2) and similar among the FHWs and MHWs. However, job satisfaction and self-efficacy are significantly different in these groups. The detailed distribution of the motivation score among these groups is presented in Table 4.

**Table 4:** Mean construct-wise motivation scores of community health workers in Ahmedabad, India, during 2018–19

Constructs of motivation	FHWs (mean ± SD)	MHWs (mean ± SD)	*p*-value
General motivation	2.1 ± 0.6	1.9 ± 0.5	0.099
Burnout	2.5 ± 0.9	2.3 ± 1.2	0.045*
Job satisfaction	1.2 ± 0.3	1.1 ± 0.2	0.001*
Intrinsic job satisfaction	1.5 ± 0.5	1.5 ± 0.5	0.824
Organization commitment	1.5 ± 0.5	1.4 ± 0.5	0.133
Conscientiousness and self-efficacy	1.7 ± 0.6	1.5 ± 0.6	0.007*
Timeliness	1.8 ± 0.4	1.8 ± 0.4	0.954
Personal issues	1.7 ± 0.6	1.8 ± 0.7	0.463
Overall motivation	1.8 ± 0.2	1.6 ± 0.2	0.001*

Min–max for the individual dimension under each construct was captured through an agreement scale of 1 to 4, i.e., strong disagreement (1) to a strong agreement (4)

*FHWs* Female Health Workers, *MHWs* Male Health Workers

*p < 0.05 is considered as significant and derived from two-sample *t*-test using variables with unequal variance

### Qualitative findings

Six FGDs (four among the FHWs and two among the MHWs) were conducted across two zones of the city. On exploring the current challenges and their motivation for becoming OHAs, the opinions were clustered on the individual, community, and health system levels based on the thematic analysis.

#### Individual level

The current job activities and work profile of the FHWs were to implement most of the national health programs like maternal child health, non-communicable diseases, or immunization. Although they are the backbone of the health system at the grass-root level, they felt demotivated due to several reasons. One of the primary reasons might be the absence of appreciation of their dedication by neither the employer nor the community. In contrast, the MHWs are under fixed-term salaries, and they are bound to be transferred to other departments within the city municipal corporation, indicating their lack of consistency in the current role. The low appreciation from the community also remained the same for the MHWs.

*“Our name is ‘ASHA’ (in the vernacular language it means Hope!), but we do not have any ‘ASHA’ (in the vernacular language it means also Expectations), they do not appreciate us, ASHA has no any appreciation”* (FHWs-FGD-3)

*“The problem is we are not working for the malaria department only, right now I am working in the malaria department, but I may get transferred to some other department within a few months. Like I was working in the solid waste management department before current assignment”* (MHWs-FGD-2)

In addition, both of these workers perceived more motivation when they have been involved in larger team activities like the last outbreaks of swine flu or bird flu. Most of these workers worked extensively during the outbreaks with or without formal training. Apart from routine work, FHWs also evinced working on the implementation of any new public health programs or piloting new interventions. They are also working with school-teachers in school health programs and some of them are also involved in mass sanitation campaigning (i.e., Sabarmati River cleaning). This indicates the multidisciplinary working culture of FHWs compared to that of the MHWs.

#### Community level

The community members’ support is a major driving force for these FHWs; they felt motivated to work hard when the community accepted them. There was mixed opinion documented for the community perceptions. Although the appreciation was low for the FHWs in most cases, most of them mentioned a positive reception by community members, from which they gain goodwill and recognition. However, some CHWs reported adverse reactions from the community while disseminating their daily routine. This might be one of the other contributing factors for the low motivation among these CHWs.

*“…..we feel proud that we are doing some good work, we feel good as they listen to us if we don’t go then they call us and tell that why we did not go there, even if we don’t go for a single day than also, they ask for us, they miss us!”* (FHWs-FGD-1)

*“…..in field people still do not understand, they think we are a beggar and came for begging something, so they use to treat us like a beggar and say ‘aage jao’ means go to next door”* (FHWs-FGD-2)

*“People do not cooperate with us! If we go for fogging in the morning, they ask to come in the afternoon, and when we go in their time, then the houses found to be locked and if we request to access to a rooftop or the water tank, they don’t allow us nor follow our any instructions”* (MHWs-FGD-1)

#### Health system level

FHWs are prime actors at the grass-root level with the multidisciplinary working culture for various health programs. Due to the inception of new programs, the activities are increasing tremendously among the FHWs, which sometimes resulted in non-scheduled work. In addition, failure to receive the financial incentives due to the non-completion of tasks or unavailability of data forms a vital system challenge. The primary issue remained the incentive-based payment system. Some of them mentioned that introducing a fixed payment for a package of services would increase their motivation for their work. There were no such system-level challenges documented by the MHWs.

*“We don’t have any fixed work schedule, they (superiors) give us diverse fieldwork if it is from the health department than ‘okay’, but it’s not like that. Today they tell to do this and next day anything else, everyday new work.”* (FHWs-FGD-1)

*“Even though all ASHA workers are working more or less the same, but do not get equal incentives, someone has more population so earning more and someone has not that much population so not getting that much. Even if we work during an outbreak, it was free; we did not get any extra incentives for that.”* (FHWs-FGD-3)

*“At present, we do not have fix pay, we people are doing work on incentive, we will get incentive according to completion of our task, the problem is if we have started any work and couldn’t complete it because of the patient side problem than we will not get the incentive for that. For an example of immunization, we have worked from the first dose of vaccination and in case if a patient would not ready to get measles dose or patient had migrated so, in that case, we would not get incentive even though we worked for rest all.”* (FHWs-FGD-2)

#### Motivation for becoming OHAs

Although FHWs have low motivation scores, certain factors documented might increase the motivation of FHWs. One aspect is confidence in what they do, and another is financial, which might motivate them to take on the additional task of OHAs. Some of them voiced concerns about the additive task from the community perspective, i.e., the opposite gender might not respond well. In addition, the acceptance of new tasks produced a concern as most of their current time is spent on data documentation. Most FHWs indicated that if the new assignment generated additional incentives, they would be pleased to do so. Therefore, an incentive package is the most important driver for the FHWs to become OHAs. In contrast, the concerns of the MHWs are mostly operational rather than financial. MHWs were found to be least concerned about the financial matters, as they are on fixed payroll as a salaried employee. MHWs have also produced similar concerns except for financial matters. Further, some of the MHWs refused to consider these additional responsibilities.

*“We (ASHA) people were entered in reproductive child health care, that time we didn’t know anything, gradually family planning, vaccination, now non-communicable diseases, yoga many more we are expert, now you can send us anywhere, we can do everything”* (FHWs-FGD-3)

*“Whether we get an incentive or not, but we always do all work for goodness of our area, all people do not think like that if incentive will be more than we will work more dedicatedly”* (FHWs-FGD-2)

*“There should be a specific day for that, and it should be merged with your routine work so you can work in between and instead of two different reports it should go at one place so whoever wants to share about their field they can”* (MHWs-FGD-1)

*“….first of all, we don’t have time. We already have our routine work which we have to finish as per the deadline”* (MHWs-FGD-2)

In addition, both types of workers have expressed their interest in proper training and skill development in the domain of OH, as this is entirely new for them. They have also requested vigorous handholding training and practices across the domains of OH. On the one hand, one group proposed that OH activities should happen on a specific day of each week (like currently Mamata day, a day for maternal/childcare); on the other hand, another group proposed OH activities need to be integrated into their daily routine. In summary, promoting MHWs as OHAs requires more stringent top-down directives while FHWs require additional financial incentives to act as OHAs.

## Discussion

OH’s operationalization is highly dependent on the development of intersectoral collaboration strategies among all the relevant stakeholders at the global, national, and local levels [41–43]. However, this intersectoral collaboration until now is an elusive paradigm [44, 45], especially at the grass-root level of implementation. This might be attributed to a lack of health system research on identifying potential actors at the grass-root level who could act as OHA. The speculative role of an OHA at the grass-root level could be direct engagement in disease control, identification of potential hazards, risk mitigation, and early recognition across the interface of human–animal–environment and overall promotion of health and well-being for all. Thus, an OH activist would not work only as a bridge between the community and the system, but also have an imperative role in reporting to different authorities responsible for diverse risk management. The potential OHAs in a local setting would be highly beneficial in operationalizing OH activities and in understanding the local challenges and community strengths.

This case study highlighted the current zoonotic disease awareness and certain activities among the CHWs and explored their motivation for becoming an OH activist in the near future. Although the overall motivation of the studied CHWs was found to be poor, they still provided themselves with positive feedbacks to act as OHA, if certain prerequisites are fulfilled. Measures like financial incentives, structured reporting patterns, assignment of clear roles and responsibilities have to be introduced before CHWs to accept the role of OHAs. Evidence indicates the importance of social recognition [46, 47] and fair monetary incentives [37, 48] for FHWs, which was also reflected in both the quantitative and qualitative findings of this study. A multi-stakeholder perspective study on the work performance of ASHAs by Sharma et al*.* [49] documented professional factors such as training and job security strengthening that would improve their performance, which was also documented in the health system-level qualitative findings of this study. Given the low density of the MHWs compared to the FHWs in India [50], the FHWs have an advantage in being considered OHAs. A systematic review found that the health system of LMICs is demotivating to the CHWs rather than motivating them to improve their performance [51], which indicates the strong need for reforms of the health system to strengthen the motivation of the health workforce. As per our observation, FHWs were more motivated to take on additional duties compared to the MHWs. However, specific financial incentives would be essential if they would be promoted to OH activists. Other studies from eastern and northern India also highlighted the importance of financial incentives to increase the level of motivation among the FHWs [37, 46]. Given the issues and challenges, some of India’s state governments started to formalize the payment to FHWs as a monthly salary rather than incentives, which is one of the most welcome steps towards improving their motivation. In addition, the intervention studies on enhancing the motivation of CHWs recommend interdisciplinary actions, such as cross-cutting approaches, training, supervision, incentives, career development, and ownership [52, 53]. While the OH activities are drawing attention to the cross-cutting approaches with its interdisciplinary nature, these might attract the targeted FHWs in building and developing more satisfaction from their work performance.

As the current OH operational strategies emphasize the education and training programs including the interdisciplinary research collaborations [41], the scope needs to be extended to train these CHWs to become OHAs. Although CHWs were trained for the selected zoonoses or participated in the zoonoses campaigning in the local setting, they urged for more intense OH training before being captivated as OHAs. Although the evidence already indicates the involvement of CHWs in pandemic control [54] and their multidisciplinary roles in infectious disease control [55] and surveillance [56], CHWs urged for more OH training on multidisciplinary teamwork. Thus, the OH training should strengthen social and leadership skills, as well as training on coordinating roles in a multidisciplinary team along with the subject knowledge. Similar factors have also been prioritized in previous research, especially when these CHWs were targeted as change agents at the community level [57, 58]. Therefore, OHA’s proposed role also needs to be envisaged in a similar pattern while promoting the FHWs as OHAs in the study setting. Despite mentioning the financial incentives as a requirement for the FHWs and as a driving force compared to the MHWs (as they were on the payroll), they are certainly not sufficient for turning the FHWs into OHAs or explaining their motivation.

When this case study recommends considering FHWs as future OHA with specific financial incentive packages, this is based on their advantages in their reach of presence, current multidisciplinary working culture, acceptance of the new health programs, higher awareness about zoonoses and current practices, and last, but not least higher motivation score as compared to the MHWs. In India so far, there have been no plans to establish a liaison between animal and human health care services at the lowest level, i.e., in the communities. Here, the OHA’s potential role could be disease reporting to the human and animal health system simultaneously while also spreading awareness about zoonoses, promoting the health of animals and humans through early detection of hazards and risks. These are no more than speculative roles and responsibilities of OHA. Policy challenges include deciding the amount for incentives, improving their motivation for OH and other health services, and taking organizational structural barriers into account.

### Limitation

This study has certain limitations: first, data collection was limited to only two zones of the city, and two types of CHWs were investigated in this study. Second, the other relevant sectors of OH need to be investigated for the presence of such community actors and recommended to test their motivation for becoming OHA. The third concern would be that approximately half of the FHWs did not respond to this study invitation, which might hint to exclusion due to educational level, social standing, or location and have had an implication on the perceptions generated herein. Therefore, there is a probability that the results might have emphasized some aspects that could restrict the generalizability of these study findings to a broader setting. Fourth, there might be other systemic factors that were not studied here and might directly or indirectly impact the CHWs and their motivation to be OHAs. Therefore, future research should consider these limitations and conduct similar studies in India’s diverse geographic settings prior to the finalization of policy recommendations.

## Conclusion

This case study highlighted the different awareness levels of selected zoonotic diseases and preventive practices among the CHWs. In addition, the overall motivation was found to be low, and most of them expressed a feeling of “burnout” in their current schedule, which needs to be accounted for during the implementation of any health programs. There were several advantages of promoting FHWs to future OHAs at the community level documented in this study, including their reach in presence, higher awareness about the selected zoonotic diseases except for brucellosis, reach in their current practices, current multidisciplinary working culture, and overall higher motivation as compared to the MHWs. However, specific measures like improving their social and institutional recognition, additive financial incentives, and top-down directives with structured guidelines need to be considered for improving their motivation as documented in the study. In addition, FHWs also emphasized gaining more training on social and leadership skills in addition to the subject matter training. FHWs could potentially serve as OHA if all identified challenges (primarily the provision of financial incentives and clear top-down guidance) are addressed before the time of commissioning them. Although this study also documented multiple systemic factors influential in shaping the OHA role outside the OH context, we recommend increasing the scope and the geographic context to understand the dynamics of the health system and account for the decisive factors beyond the OH area.

## Data Availability

Data from this study will be available at the Center for Development Research (ZEF), Bonn, Germany, after the completion of this study. Researchers who meet the criteria for access to confidential data are encouraged to approach Dr. Timo Falkenberg, Coordinator Fortschrittskolleg ‘One Health’, Center for Development Research (ZEF), Bonn, Genscherallee 3, 53113 Bonn, Germany. Email: falkenberg@uni-bonn.de.
